# Involving people with diabetes and the wider community in diabetes research: a realist review protocol

**DOI:** 10.1186/s13643-015-0127-y

**Published:** 2015-11-04

**Authors:** Janet Harris, Marit Graue, Trisha Dunning, Johannes Haltbakk, Gunhild Austrheim, Nina Skille, Berit Rokne, Marit Kirkevold

**Affiliations:** School of Health and Related Research (ScHARR), University of Sheffield, Sheffield, UK; Faculty of Health and Social Sciences, Bergen University College, Bergen, Norway; Department of Paediatrics, Haukeland University Hospital, Bergen, Norway; Centre for Nursing and Allied Health Research, Deakin University and Barwon Health, Geelong, Australia; Diabetes Association, Oslo, Norway; Department of Global Public Health and Primary Care, The Faculty of Medicine and Dentistry, University of Bergen, Bergen, Norway; Department for Research and Development, Haukeland University Hospital, Bergen, Norway; Department of Nursing Science, Institute of Health and Society, University of Oslo, Oslo, Norway

**Keywords:** Diabetes mellitus, Health services research, Consumer participation, Patient participation, Community-based participatory research, Realist review

## Abstract

**Background:**

Patient and public involvement in diabetes research is now actively encouraged in different countries because it is believed that involving people with experience of the condition will improve the quality and relevance of the research. However, reviews of patient involvement have noted that inadequate resources, patients’ and communities’ lack of research knowledge, and researchers’ lack of skills to involve patients and communities in research may present significant contextual barriers. Little is known about the extent of patient/community involvement in designing or delivering interventions for people with diabetes. A realist review of involvement will contribute to assessing when, how and why involvement works, or does not work, to produce better diabetes interventions.

**Methods/design:**

This protocol outlines the process for conducting a realist review to map how patients and the public have been involved in diabetes research to date. The review questions ask the following: How have people with diabetes and the wider community been involved in diabetes research? What are the characteristics of the process that appear to explain the relative success or failure of involvement? How has involvement (or lack of involvement) in diabetes research influenced the development and conduct of diabetes research? The degree of support in the surrounding context will be assessed alongside the ways in which people interact in different settings to identify patterns of interaction between context, mechanisms and outcomes in different research projects. The level and extent of the involvement will be described for each stage of the research project. The descriptions will be critically reviewed by the people with diabetes on our review team. In addition, researchers and patients in diabetes research will be asked to comment. Information from researcher-patient experiences and documents will be compared to theories of involvement across a range of disciplines to create a mid-range theory describing how involvement (or lack of involvement) in diabetes research influences the development and conduct of diabetes research.

**Electronic supplementary material:**

The online version of this article (doi:10.1186/s13643-015-0127-y) contains supplementary material, which is available to authorized users.

## Background

In the last 15 years, there has been a huge amount of research conducted on how to promote professional and patient competencies in managing diabetes. There is some evidence that interventions that are modified to the personal and social background of participants can improve knowledge of diabetes and healthy lifestyles and promote glycaemic control [1]. Recently, professional organisations have noted that researchers need to be better informed about the needs of the target population so that they can ‘tailor’ interventions. Consequently, the National Standards for Diabetes Self-Management Education in America emphasise needs assessment as an essential standard in education to promote diabetes self-management [2]. Strategies for ensuring appropriateness include learning about previous experiences of receiving diabetes interventions and using the learning to tailor interventions [3]. The nature and level of patient involvement in tailoring, however, is seldom reported as the phenomenon of interest.

Interventions that are done ‘to’ or ‘for’ people by researchers may not empower people with diabetes to manage their condition. If people are involved in designing and studying an intervention, however, they may feel better able to manage chronic conditions [4]. Some definitions for involvement assume that research should be informed by the perspectives, interests and values of people with the condition throughout the research process. Involvement can occur at the stage of selecting research questions and continue to the dissemination of research results [5]. The importance of involving communities and people in the design and delivery of interventions has been recently affirmed by Liu et al. [3]. They found strong evidence that collaboration with local and respected leaders, institutions and professional organisations with expertise in experiences of ethnic groups was essential to recruit participants to research studies and design appropriate interventions. A similar review of the research is needed to determine whether active involvement produces more appropriate and acceptable interventions specifically for people with diabetes.

### Conceptual framework

Opportunities for involving people with diabetes may occur throughout research design and implementation or be used at selected stages of the research (Table 1).

**Table 1:** Opportunities for user involvement in designing and conducting research

● Pre-research: identifying research priorities, suggesting research ideas, commenting on usefulness of the research
● Proposals: joint grant holders or co-applicants on a research project; consultants who comment on the wording of proposals or the wording of ‘lay language summaries’; feedback on the perceived relevance and utility of the proposed outcomes from a patient perspective
● Research design: participate in design of the intervention for people with diabetes, review the language and user-friendliness of questionnaires, commenting and developing patient information leaflets and printed materials
● Recruitment: contribute to ideas for ensuring good recruitment and participation in the study, feedback on patient consent forms and project information sheets
● Data collection: participate in data collection as lay researchers undertaking interviews with research participants as user and/or carer researchers
● Analysis: co-analyse data—this may include analysis of qualitative themes and identification of areas where additional clarification or further sampling is needed
● Dissemination: ensuring findings are circulated and presented to local communities, patient groups, organisations

Modified from Lindenmeyer et al. [13]

### Levels of involvement

At each stage, different levels of involvement may occur. There may be minimal involvement when specialised technical knowledge is needed. For example, when deciding upon a plan for statistical analysis, people may simply be informed of the approach that will be taken. Maximum involvement, on the other hand, may occur when academic researchers lack experiential knowledge. For example, where there is little experience of recruiting people from vulnerable groups to a study, patients and/or community members may take control of developing and implementing recruitment strategies. The nature and extent of involvement therefore needs to be considered as an essential part of an involvement ‘matrix’ that also includes the researchers’ perspectives on sharing control, and involvement at various project stages [6].

The underlying theory for involvement posits that if service users are actively involved in prioritising, commissioning, undertaking, communicating and using research, then their unique perspectives will improve the quality and relevance of the research [7]. As can be seen from the matrix of involvement presented above, successful involvement is complex, requiring a context where people feel supported and motivated to contribute. The context may need to include provision of training to participants to develop understanding of the research process, training to researchers to develop skills in interacting with members of the public, alongside support from funders. If the context is supportive, various mechanisms may be triggered such as participants experiencing increased confidence in interacting with researchers, and researchers being more willing to allow projects to be co-designed. To date, very few research studies have explored the process of involvement in this way. Although there is ‘evidence that patient and public involvement can have positive impact on research, enhancing the quality of research and ensuring its appropriateness and relevance’, the evidence justifying the value of involvement remains weak [6, 7]. Further, potential benefits may be cancelled out by challenges encountered when trying to involve people, which include managing short timescales, negotiating disagreements about appropriate research design, recruiting people to help with the project, maintaining participation during data collection and analysis, and cost of involvement.

Although governments and policymakers are interested in routinely involving patients and the public [8, 9], there are indications in diabetes research that funded projects do not reflect the issues that patients and carers feel are priorities for research [10]. A scoping of the literature indicates that some diabetes projects have routinely involved patients at various stages of designing and delivering interventions, but the extent of involvement or the specific challenges encountered in diabetes research have not been systematically reviewed. Involvement in developing the intervention may have a positive (or negative) effect in terms of ability to benefit from the intervention, but no research has explored the relationship between involvement, empowerment and ability to self-manage diabetes. The effectiveness of involvement, therefore, cannot be reviewed until the process of involvement and its relative contribution to research is explored. A realist review will enable us to explore whether involvement can lead to development of better interventions. Realist reviews draw on diverse sources of information to examine the resources available in different settings (context) and the way settings influence people’s attitudes and actions (mechanisms) in relation to desired outcomes. They are also useful for documenting changes in the process over time. The approach will enable us to explore how strategies for involvement work in particular contexts but not in others. Increased or decreased involvement can also be documented across stages of research, which may capture the development of involvement skills and evolution of working relationships. By looking across diverse contexts, we aim to develop explanations for how researchers, people with diabetes and the surrounding communities with varying levels of skill and experience have interacted to create appropriate interventions.

The findings from the review will inform a programme funded by the Norwegian Research Council called DiaHealth (http://prosjekt.hib.no/diahealth/). Within the DiaHealth project, we are developing, piloting and evaluating a web-based intervention in primary care that aims to promote diabetes self-management and empowerment. The study involves service users in the development of web-based support for self-management. The purpose of this study is to develop new knowledge on how the use of web-based follow-up can improve HbA_1c_ and self-management of type 2 diabetes in adults. The realist review will produce both explanations for challenges to involvement and solutions, which can be used to inform intervention development in DiaHealth, as well as inform evaluation design.

### Objectives and focus for the review

The objectives of this review are to map the existing literature on patient involvement in diabetes research and to develop a preliminary theory of the contexts that enable involvement in diabetes research by people with diabetes, carers and the wider community.

### Review questions

- RQ1: How have people with diabetes and the wider community been involved in setting priorities, designing and conducting diabetes research?
- RQ2: What are the main characteristics of the process that appears to explain the relative success or failure of involving people with diabetes and the wider community in diabetes research?
- RQ3: How has the involvement (or lack of involvement) in diabetes research influenced the development and conduct of diabetes research?

## Methods/design

PROSPERO registration for the review was sought but was not required because the review was classified as a methodology review—one which focuses solely on the research process rather than health outcomes. The review team is comprised of a subset of researchers in the DiaHealth project, patients from the Diabetes Association and realist reviewers. People on the team are implementing policies for improving diabetes care, delivering diabetes services and receiving diabetes services. They are also working with a larger group of people in DiaHealth who are conducting and participating in research studies. These multiple perspectives should promote the development of understanding about how involvement in designing interventions is influenced by the surrounding context. The team has co-developed the protocol and will use their connections to disseminate information throughout the review for discussion.

### Search strategy

Our first search will ‘map the territory’ of involvement in diabetes research. Key terms for diabetes, insulin resistance and glucose intolerance will be used to identify people with diabetes or at risk for developing diabetes. Terms for involvement—such as consumer panel, advisory board, participation, and engagement in research—will be taken from previous systematic reviews of patient and public involvement in research [7]. Terms for community-based participatory research will also be used from a previous review [11]. A recent review and synthesis of concepts related to involvement in health research noted that involvement is used across different types of research, ranging from clinical trials to community-based participatory research [12]. CBPR is increasingly being used because there is growing recognition that health systems need to partner with communities to gain greater involvement in health [13]. Health databases (Medline, CINAHL, EMBASE) will be searched in the first stage of the scoping, with no limitations on date, language or study type.

Our previous experience with realist reviews has shown that single articles often do not report all of the relevant information on context, processes, interactions or mechanisms in relation to outcomes. Further, we know from previous experience with community-based participatory research that study designs can take a number of years to evolve because trust needs to be developed with the communities who would be supporting the intervention. We therefore need to systematically identify all of the documents for each project, which will maximise our chances of obtaining a rich description of the process. This can be done by using an approach called ‘cluster’ searching [14]. Cluster searching combines a search on members of the research team with references within relevant articles to obtain all refereed journal articles alongside grey literature reports for a project. In a previous review, a Google Scholar search on author, combined with use of backwards and forwards citation analysis via the Science Citation index, produced all related documents for each project. Reviewing an entire cluster will enable us to identify the different dimensions of involvement, chart involvement at various stages and document perceptions about challenges and progress. We will be able to assess how involvement is influenced by the surrounding context, how involvement increases or decreases over time, and the longer-term benefits of involvement.

### Study inclusion criteria

Studies will be selected in the first instance if they satisfy the criteria for relevance and rigour. Relevance is defined as the pertinence of the study to the review question, and rigour is defined as whether the method used to make the outputs contain adequate information to generate data on the process of involvement [15].

The population will be defined as people with diabetes or pre-diabetes, carers of people with diabetes and people from organisations that represent people who use diabetes services. Involvement in diabetes research is defined as participation in the design and/or conduct of research either by informing or advising a research organisation or a specific research study. These criteria are based on the INVOLVE definitions for public involvement in research [4]. Descriptions of involvement will be contained within documents, but in most cases, will not be the focus of the study. The quality of information will be judged on the claims made about the process of involvement, completeness of the description, number of participants and how data was collected and analysed. This will be done after data is extracted and prior to developing explanations for involvement.

**Fig. 1:**
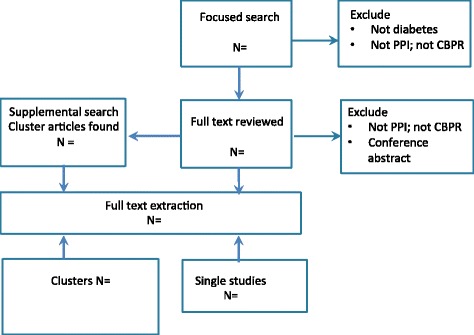
Flow chart for study selection

We will keep a record of the types of data sources identified and included using a modified version of a PRISMA flow chart (Fig. 1) [16].

### Data extraction

There are several frameworks that can be used to classify patient and public involvement; however, to our knowledge, none have been used as data extraction tools. We will therefore pilot three different frameworks in the first stage of our data extraction to identify which framework is the most appropriate or whether elements of the three different frameworks should be combined. The first, a framework by Telford et al. [12], focuses on the skills needed to participate as well as on the communication and reporting process (see Additional file 1). The second, by Shippee et al. [17] presents involvement by the stages of designing, implementing and disseminating research (see Additional file 1). The Shippee framework integrated different involvement concepts from community-based prevention research aimed at developing clinical guidelines [18–22]. The third conceptual framework proposed by Popay [23] categorises the overall level and type of participation from the perspective of community engagement (see Additional file 1).

Characteristics of included studies will be documented, including setting, research design, participant characteristics, methods, findings and authors’ conclusions. The process of involvement will be extracted including when involvement occurred, who was involved, how the process was facilitated and what people did at each stage. We will categorise projects as having ‘successful’ outcomes if involvement was seen as contributing to designing and implementing the research. Descriptions of the context surrounding the study will be compiled, such as resources available (training, funding, supervision), researchers’ knowledge of involvement and skills, support given to participants and time allotted in the project for involvement. Reflections on the process will also be extracted in order to identify mechanisms such as attitudes toward involvement, opinions on whether involvement makes a useful contribution, motivation to be involved and challenges encountered. How supportive contexts trigger these mechanisms to produce successful involvement outcomes will be described for each project separately.

Each team member will work with a subset of the included documents. Rigour will be ensured by having a second reviewer (JH) check the data extraction and the context-mechanism-outcome descriptions. The descriptions will also be critically reviewed by the people with diabetes on our review team and brought to the researchers and patients participating in the DiaHealth project for comment.

Theories explaining the contribution of patient involvement will be found by identifying reviews and primary studies that describe the potential value of patient involvement in health research, and identifying studies that specifically apply theories of involvement to the design and conduct of diabetes research. Furthermore, discussions with stakeholders who have experience of involving users in diabetes research will be conducted. The sources for these theories will include electronic databases of primary research, grey literature such as reports including descriptions of research design and implementation, guides for training lay people to contribute to research [24] and evaluate involvement [25], and researchers within DiaHealth who have experience of involving users in research.

### Data synthesis and theory building

We will work with researchers and participants in the DiaHealth team to co-produce a description of what works, for whom, and at what point in time when involving people with diabetes in research.

This will be refined and tested through a participatory process where the review team presents the emerging theory to researchers and service users in the DiaHealth project. We will ask them to reflect on whether the experiences of involvement described resonate with theirs. Participants may agree or may identify important components of involvement based on their experiences which were poorly reported in publications. From previous experience [26], we expect this iterative process to contribute to building theory in terms of what works, for whom and in what circumstances to facilitate involvement in diabetes research. Patterns describing the relationship between type of involvement and research process and outcome will be identified by the different stages in diabetes research projects. As these patterns become clear, we will conduct additional searches to identify how the explanation maps to more general theories for involvement. These may not only be found within the field of health, but may also exist in fields such as community development and citizen or civic involvement. The information from this process will be used to develop a mid-range theory—an explanation for how and why involvement (or lack of involvement) influences the development and conduct of research. We will report the findings according to the publication criteria outlined by the RAMESES publication standards [15].

The middle-range theory will be used to explain why and how patient involvement can produce more feasible and appropriate interventions, and help explain why interventions have differing degrees of success in different contexts and populations. We believe these explanations will be useful for diabetes researchers who are currently designing interventions and those who are looking for reasons for the relative ineffectiveness of existing interventions. The review will also contribute to our understanding of theory-based approaches to evaluating involvement across health topics.

## Discussion

The discussion section of the realist review will briefly summarise the findings in terms of their implications for future research. The quality of reporting on the process of involvement will be considered, providing examples of good reporting practice that we identify from the literature [27]. From our previous experience and prior involvement reviews, we would expect the discussion to cover (but not be limited to) issues of combining expert provider/researcher knowledge with experiential knowledge of patients and carers, and challenges of involvement by stage of research, in terms of skills, timing and resources. Constraining contextual factors, such as short funding application deadlines and funder expectations for delivery, will be presented and if possible innovative solutions offered from the literature. We will identify how mechanisms of involvement can explain the success of different interventions in diabetes care. The strengths and limitations of the realist review will be considered, as well as future research directions. We will critique the methods for the realist review and the strength of the emerging theory for effective involvement in diabetes research. The emerging theory for what makes good user involvement in diabetes research will be compared with similar research both in diabetes and in the broader fields of public-patient involvement and community involvement across a range of health topics. This can inform further research to understand the mechanisms at play for the development of health care interventions that will benefit people with diabetes and their families.

### Dissemination

Realist reviews aim to provide explanations that can be used to inform policy and practice. Our review will aim to inform practice during the duration of the DiaHealth project by consistently involving the project researchers and service users in considering emerging findings as well as contributing to theory building. As the aim of the project is to inform policy makers, preliminary findings and the final theory of involvement will be presented to them for review and comment, along with specific recommendations based on current practice for when, how and who to involve in future diabetes research.

## Additional file

Additional file 1:
**Framework for successful consumer involvement (Telford et al. [** 12 **]). Framework for patient and service user involvement (Shippee et al. [** 17 **]). Levels of participation (Popay [** 23 **]).**

## Abbreviations

CBPR: community-based participatory research
PPI: public-patient involvement in research
RAMESES: realist and meta-narrative evidence syntheses: evolving standards
