# Supplementary material for: Involving people with diabetes and the wider community in diabetes research: a realist review protocol
Source: Syst Rev. 2015 Nov 4;4:146. doi: 10.1186/s13643-015-0127-y (PMC4632468; doi:10.1186/s13643-015-0127-y)
Supplement: Additional file 1: — Framework for successful consumer involvement (Telford et al. [ 12 ]). Framework for patient and service user involvement (Shippee et al. [ 17 ]). Levels of participation (Popay [ 23 ]). [file 13643_2015_127_MOESM1_ESM.doc]

**Additional file 1**

**Initial framework for successful consumer involvement (Telford et al, 2004)**

| **Principle Indicator(s)** | **Criterion used in data extraction** |
| --- | --- |
| 1) The roles of consumers are agreed between the researchers and consumers involved in the research | The roles of consumers in the research were documented |
| 2) Researchers budget appropriately for the costs of consumer involvement in research | Researchers applied for funding to involve consumers in the research |
|  | Consumers were reimbursed for their travel costs |
|  | Consumers were reimbursed for their indirect costs (e.g. carer costs) |
| 3) Researchers respect the differing skills, knowledge and experience of consumers | The contribution of consumers skills, knowledge and experience were included in research reports and papers |
| 4) Consumers are offered training and personal support, to enable them to be involved in research | Consumers training needs related to their involvement in the research were agreed between consumers and researchers |
|  | Consumers had access to training to facilitate their  involvement in the research |
|  | Mentors were available to provide personal and technical support to consumers |
| 5) Researchers ensure that they have the necessary skills to involve consumers in the research process | Researchers ensured that their own training needs were met in relation to involving consumers in the research |
| 6) Consumers are involved in decisions about how participants are both recruited and kept informed about the progress of the research | Consumers gave advice to researchers on how to recruit participants to the research |
|  | Consumers gave advice to researchers on how to keep participants informed about the progress of the research |
| 7) Consumer involvement is described in research reports | The involvement of consumers in research reports and publications was acknowledged |
|  | Details were given in the research reports and publications of how consumers were involved in the research process |
| 8) Research findings are available to consumers, in formats and in language they can easily understand | Research findings were disseminated to consumers involved in the research in appropriate formats (e.g. large print, translations, audio, Braille) |
|  | The distribution of the research findings to relevant  consumer groups was in appropriate formats and easily understandable language |
|  | Consumers involved in the research gave their advice on the choice of methods used to distribute the research findings |

Framework for patient and service user involvement (Shippee et al, 2013)

| Stage | Sub-stage |
| --- | --- |
| Preparatory | Agenda setting and funding |
| Execution | Study design and procedures |
|  | Study recruitment |
|  | Data collection |
|  | Data analysis |
| Translational | Dissemination |
|  | Implementation |
|  | Evaluation |

Nature and extent of involvement **(Popay, 2010)**

| **Level of involvement** | **Examples relevant to diabetes research** |
| --- | --- |
| Community control | Patients and the public are co-researchers responsible for significant portions of the design and conduct of the study. They may co-direct research teams which include academic researchers; hold the budget for the project; and/or have the authority to question quality or use stopping rules if there are any questions about the conduct of the study. |
| Delegated control | Patients and the public may be made responsible for particular aspects of the project, such as maximising recruitment or reviewing patient information. |
| Co-production | Patients and the public are asked to serve as advisors to one or more aspects in the prioritisation, design, development, and delivery of an intervention and its evaluation. |
| Consultation | Patients and the public are asked to review an aspect of the project. This consultation may be a single encounter, and ways in which the information was used may not be fed back to participants. |
| Informing | Patients are recruited to a research project where there was no involvement. |
